# Risks and challenges in COVID-19 infection prevention and control in a hospital setting: Perspectives of healthcare workers in Thailand

**DOI:** 10.1371/journal.pone.0267996

**Published:** 2023-12-19

**Authors:** Monnaphat Jongdeepaisal, Puri Chunekamrai, Rapeephan Rattanawongnara Maude, Richard James Maude

**Affiliations:** 1 Mahidol-Oxford Tropical Medicine Research Unit, Faculty of Tropical Medicine, Mahidol University, Bangkok, Thailand; 2 Centre for Tropical Medicine and Global Health, Nuffield Department of Medicine, University of Oxford, Oxford, United Kingdom; 3 Faculty of Medicine, Srinakharinwirot University, Nakhonnayok, Thailand; 4 Faculty of Medicine and Health Science, University of Nottingham, Nottingham, United Kingdom; 5 Faculty of Medicine, Ramathibodi Hospital, Mahidol University, Bangkok, Thailand; 6 Harvard TH Chan School of Public Health, Harvard University, Boston, MA, United States of America; 7 The Open University, Milton Keynes, United Kingdom; Delta State University, NIGERIA

## Abstract

**Introduction:**

In hospital settings, awareness of, and responsiveness to, COVID-19 are crucial to reducing the risk of transmission among healthcare workers and protecting them from infection. Healthcare professionals can offer insights into the practicalities of infection prevention and control (IPC) measures and on how the guideline aimed to ensure adherence to IPC, including use of personal protective equipment (PPE), could best be delivered during the pandemic. To inform future development of such guideline, this study examined the perspectives of healthcare professionals working in a large hospital during the pandemic regarding their infection risks, the barriers or facilitators to implementing their tasks and the IPC measures to protect their safety and health and of their patients.

**Method:**

In-depth interviews were conducted with 23 hospital staff coming into contact with possible or confirmed cases of COVID-19, or were at potential risk of contracting the disease, including medical doctors, nurses, virology laboratory staff, and non-medical workers. This qualitative study was carried out as part of a knowledge, attitudes and practice survey to prevent COVID-19 transmission at Ramathibodi Hospital in Thailand. We used content analysis to categorize and code transcribed interview data. Existing IPC guideline and evidence synthesis of organisational, environmental, and individual factors to IPC adherence among healthcare workers were used to guide the development of the interview questions and analysis.

**Finding:**

Factors identified as influencing the use of, and adherence to, prevention measures among healthcare workers included knowledge, perceived risk and concerns about the infection. The extent to which these factors were influential varied based on the medical procedures, among other features, that individuals were assigned to perform in the hospital setting. Beyond availability of PPE and physical safety, ease of and readiness to utilize the equipment and implement IPC measures were crucial to motivate hospital staff to follow the practice guideline. Having a ventilated outdoor space for screening and testing, and interaction through mobile technology, facilitated the performance of healthcare workers while reducing the transmission risk for staff and patients. Adequate training, demonstration of guided practices, and streamlined communications are crucial organisational and management support factors to encourage appropriate use of, and adherence to, implementation of infection prevention and control measures among healthcare workers.

**Conclusion:**

This finding could help inform the development of recommendations to optimise compliance with appropriate use of these measures, and to improve guidance to reduce HCW’s risk of disease in hospital settings. Further study should explore the perceptions and experiences of health professionals in smaller health facilities and community-based workers during the pandemic, particularly in resource-limited settings.

## Background

During the multiple waves of COVID-19 transmission, healthcare workers (HCWs) play a central role in saving lives. These professionals are the driving force to achieving effective clinical management of patients during the pandemic. They are also among the population with highest risk of infection with COVID-19 due to their contacts with high-risk individuals and working environment [1]. Of the 3.45 million global deaths due to COVID-19 reported to the World Health Organization (WHO) from January 2020 to May 2021, only 6,643 were among HCWs with the true figure estimated to be much higher at around 80,000–180,000 [2]. In Thailand, suspected cases of infection with emerging infectious disease caused by SARS-CoV-2 were identified during early January 2020 [3]. In April 2020, 102 HCWs, or 4 percent of total cases at the time, were infected with COVID-19; 28 of which had been providing direct care to patients, six had close contact with other HCWs, and one had been conducting screening and triage [4]. Indigenous cases of COVID-19 were found to be minimal during the first wave [5]; however, a larger second wave occurred from mid-December 2020 onwards [6]. Subsequent waves of the pandemic in 2021, during which the number of cases increased in Bangkok and across the country [7], represented a significant rise in the burden for hospitals, with over 600 HCWs reported to be infected with COVID-19 despite receiving two doses of Sinovac vaccine [8]. Most recently, in early 2022, there has been a rise in cases and staff absences due to the Omicron variant with potential for rapid spread including in healthcare settings. With the ongoing uncertainty and possibilities of new outbreaks in the future, COVID-19 remains a dangerous infectious disease, particularly for HCWs involved in managing infected patients.

In hospital settings, infection prevention and control (IPC) programmes are crucial to reducing the risk of health care-associated infections among HCWs [9]. To protect HCWs, WHO’s guideline for COVID-19 IPC in healthcare settings recommends use of personal protective equipment (PPE), hand hygiene and IPC training [10]. To inform the identification of risk factors for COVID-19 infection, WHO has investigated the extent of infection in health care settings and risk factors for infection among HCWs [11]. A survey of HCWs in 57 countries found that an overall high level of awareness and preparedness among HCWs who received training courses during the first wave of the pandemic, with variation regarding gender, type of HCWs, and prior experience with outbreaks [12]. HCWs can provide insights into the practicalities of IPC measures, which have seen insufficient attention. To optimise compliance with appropriate use of PPE and IPC training, additional information is needed to understand the experience of HCWs during the pandemic and their opinions on how the PPE and IPC training could best be delivered. Considering the ongoing need for protection from COVID-19 and the high level of risk among HCWs, it is critical to explore the views and perceptions of this population towards their occupational safety.

Through in-depth interviews, this study aimed to examine the perspectives of healthcare professionals working in a large hospital during the pandemic regarding their infection risks, the barriers or facilitators to implementing their tasks and adhering the IPC measures. This was done to inform the development of future guidance to reduce future risk of disease among HCW.

## Method

### Study design

This qualitative component was carried out as part of a knowledge, attitudes and practice (KAP) survey to prevent COVID-19 transmission at Ramathibodi Hospital in Thailand in 2020 [13]. We used qualitative in-depth interviews (IDIs) to provide detailed information on perspectives of HCWs on their risks of infection and concerns about working in the pandemic in a hospital setting [14]. Open-ended questions and probing techniques were employed to elicit perceptions and experiences from the interview participants [15]. All interview data underwent qualitative content analysis [16]. The COREQ (COnsolidated criteria for REporting Qualitative research) Checklist was employed to report this study (see S1 File).

### Study setting

The study was conducted at Ramathibodi Hospital which is a public tertiary medical school hospital in Bangkok, Thailand. Since the beginning of the pandemic, the hospital has provided COVID-19 testing and care for suspected and confirmed COVID-19 patients. The virology laboratory at the hospital performed virology testing services for the hospital as well as other hospitals or clinics that sent their samples. An extended campus, Chakri Naruebodindra Medical Institute (CNMI), was subsequently designated to admit patients with confirmed COVID-19. Approximately 300 medical workers at the hospital were reported in July 2021 to be infected whilst the hospital provided care for approximately 1,000 COVID-19 hospitalized patients, 350 patients in home isolation and 200 others waiting to be admitted for treatment [17].

### Participants and recruitment

Participants were recruited through a combination of purposive and convenience sampling. Eligible participants included any hospital staff coming into contact with possible or confirmed cases of COVID-19, and who were at potential risk of contracting COVID-19. This included medical doctors and nurses who cared for suspected and/or confirmed COVID-19 patients in the hospital, laboratory staff who handled clinical specimens from patients with suspected or confirmed COVID-19, and IPC nurses who designed the IPC guidelines and trained hospital staff. Potential participants were identified from among those who had participated in the KAP survey [13]. They were informed about the study and invited to join the in-depth interviews either by telephone or face-to-face at the hospital. Study information, including rationale and objectives, were provided to potential participants. Location at the hospital and time of the interviews were arranged with those who provided consent. The interviews were conducted one-on-one in a quiet meeting room or other location preferred by participants, for example a bench at a communal outdoor space within the hospital. Purposive sampling was used to ensure that healthcare workers with diverse roles, experiences and age groups in the hospital were interviewed. Additional participants were identified via a snowballing technique in various medical units, hence, we were able to reach non-medical staff who did not come in contact with people (possibly) having COVID-19 and thus may not have been captured in the initial phase. The total number of interviews was determined by a point of saturation whereby no further novel information was forthcoming from subsequent data collected [18]. The data analysis process was conducted iteratively with interviews, which enabled the researchers to determine the point of saturation. Although the total sample size was not predetermined, 15–20 participants were estimated for the interview data to be theoretically saturated.

### Study procedures and data collection

An interview guide was developed based on a rapid review of literature on barriers and facilitators to HCWs adhering to infection prevention and control guidelines in healthcare settings [19]. This review was completed at the beginning of the pandemic and synthesised research relating to other respiratory infectious diseases to inform IPC guidelines in the context of COVID-19. The review employed ‘Theoretical Model to Explain Self-Protection Behavior at Work’ as a framework to investigate perceptions of HCWs and factors affecting their adherence to IPC [20]. We also reviewed the WHO’s 2020 interim guidance (updated version July 2021) on infection prevention and control during health care when coronavirus disease (‎COVID-19)‎ is suspected or confirmed [10] intended to inform IPC staff in order to minimize COVID-19 transmission in hospital settings among healthcare workers and patients. From these key documents, we defined three broad categories for the interview guide: organization, environment, and individual (see S2 File).

The guide was semi-structured, and designed in English and translated to Thai, and was piloted with the first four recruited participants as part of the initial interviews to check for any miscommunication. In addition to the interview questions, the interviewer was asked to summarized the key interview responses to the participants to validate and capture any important data that were missed during the interviews.

IDIs were conducted in person in Thai by a single female interviewer and social scientist (MJ) with the support of one female and one male medical professionals (RRM and RJM) on the technical knowledge about COVID-19, infection prevention and the hospital system. The interviews took on average 45–90 minutes. The interviews began with a general discussion of their work activities during the COVID-19 pandemic and continued with key topic areas and lists of suggested questions. The interviewer did not have prior contact with and was not known to the participants but was introduced to the potential participants through established contacts with two to three hospital staff and initial participants. The interviews were conducted from June to September 2020, in parallel with the KAP survey data collection.

### Data processing and analysis

After participants gave their consent, interviews were audio-recorded and subsequently transcribed verbatim and translated to English or summarised into detailed notes by two researchers (MJ and PC). The translated transcripts and notes were imported into NVivo version 12 (QSR International Australia) for qualitative content analysis [16] by MJ. An initial codebook was developed using established categories based on the original research questions and revised as new themes emerged from reoccurring data and during debriefs where the codebook was discussed and refined. The codebook was flexible, and the codes were reassessed during data collection and revised according to the emergence of novel themes. Transcripts were read several times and coded line-by-line using an inductive and deductive approach (by MJ and PC): the codebook used was initially based on the main research topics. Subsequently, during the process of coding, themes that emerged from the data were incorporated into the codebook (see S3 File).

### Ethical considerations

Ethical approval was obtained from the Human Research Ethics Committee, Faculty of Medicine Ramathibodi Hospital, Mahidol University (approval reference: COA. MURA2020/828, 22 May 2020). All respondents provided written informed consent to be interviewed and audio-recorded. The interview data were encrypted and contained no personally identifiable information in the recording or transcription itself or the file name. Respondents were informed that they have no obligations to participate and there will be no consequences for them, should they decide not to participate; their decision to participate, or not, will be kept confidential. Interviews were conducted in a quiet location at their places of work. The interviewer (MJ) was granted permission from the hospital authorities to access the hospital during the data collection period.

### Findings

The findings presented are based on individual in-depth interviews with 23 healthcare workers in various roles including nurses, medical doctors, and laboratory technicians. **Table 1** summarises the characteristics of the interview participants. **Table 2** shows interview excerpts on facilitators and barriers to healthcare workers’ adherence with infection prevention and control (IPC) guidelines

**Table 1:** Characteristics of interview respondents. F = Female, M = Male, OPD = Outpatient Department, IPC = Infection Prevention and Control, ER = Emergency Room.

Sex	Age range (years)	Type of healthcare worker/hospital department
F	20–29	Nurse/OPD
F	20–29	Nurse/OPD
F	40–49	Nurse/OPD
F	40–49	Nurse/OPD
F	40–49	Nurse/OPD
F	30–39	Nurse/OPD
F	40–49	Nurse/OPD
F	20–29	Nurse/OPD
F	30–39	Nurse/OPD
F	30–39	Nurse/IPC
F	30–39	Nurse/IPC
M	30–39	Scientist/Virology Laboratory
M	20–29	Scientist/Virology Laboratory
F	40–49	Scientist/Virology Laboratory
F	40–49	Medical technician/Virology Laboratory
F	50–59	Senior scientist/Virology Laboratory
F	50–59	Senior scientist/Virology Laboratory
M	50–59	Medical doctor/Infectious Disease
F	30–39	Medical doctor/Anaesthetist (ER)
F	30–39	Medical doctor/Anaesthetist (non-ER)
M	40–49	Medical doctor/Anaesthetist (non-ER)
M	30–39	Non-medical/Auxiliary staff
M	40–49	Non-medical/Auxiliary staff

**Table 2:** Interview excerpts on facilitators and barriers to healthcare workers’ adherence with infection prevention and control (IPC) guidelines; topics and definition adapted from Hougton et al. 2020 [19].

Topics	Definition and key points	Excerpts from interviews on facilitators or barriers
**Organisational factors**		
Safety climate	What management support is needed to create a safe workplace (workload, staffing)	*“[At the virology lab] Everyone has their own assigned tasks to work on in addition to our routine work*. *Apart from this*, *there’s also a meeting during emergency outbreak where we decide with the department head how to manage workload during the outbreak*. *We will separate our work in sections where some amplify the sample*, *some inactivate the virus*, *etc*. *Everyone will practice their section until they become quite proficient at it*, *then all the staff switch position in a loop*, *thus allowing this unit to work for 24 hours every day including holidays… To be honest*, *it is quite hard to do this because we don’t really know when the samples will come in*, *and how many there will be so this process is quite tricky*.*” (IDI-HCW001 female)* *“Mostly in our laboratory we have a closed operating system*, *which means the only way for hospital staff to interact with us is to send samples or specimens*. *For example*, *there are a specific cleaning staff who were responsible for collecting the infectious waste*. *Or when the other laboratory delivered us their samples*, *we have a biosafety cabinet to store them*, *rather than meeting the delivery person directly*.*” (IDI-HCW002 male)* *“I think the [infection] risk is all over the hospital … starting from the carpool officer*, *medical record nurses*, *nurses*, *laboratory staff*, *cleaners*.* *.* *. *It’s like a flow where everyone could be affected*. *However*, *we do have a risk management unit to examine whether each area is contaminated*, *or not*. *For example*, *every time a patient uses a wheelchair*, *that wheelchair will need to be cleaned and sterilized*.*” (IDI HCW011 female)*
Communication of IPC guidelines	How best to communicate the guidelines	“*In terms of communication*, *we really needed collaboration from many departments*. *Medical doctors could explain to staff about the disease and its transmission route … infection control nurses would give training on how to use prevention measures*. *Fellow doctors would need to demonstrate how to do a swab test*. *We also needed to communicate with laboratory staff about how the samples will be taken and they informed us about how to transport them … training was clear and well-delivered so that most nurses are able to do a swab test now*.” *(IDI HCW012 female)*
Availability of training programmes	What the training needs are and who should deliver the training	“*At the infection control unit*, *we normally provide training or suggestions to staff and not always by the book*. *We also observed their working environment and asked about their needs … to make them feel less worried*, *make ourselves part of their work*, *that we were in this together and also let the staff participate in adapting the guideline for their own use*” *(IDI-HCW017 female)* *“There is a yearly plan for all of us to train … our supervisors would assign the slots … which turned out quite well but for the very busy individual*, *the training could delayed … I enjoyed the training*, *to keep updated about the equipment and technologies*. *These sessions largely supported our work because if we do not follow any news or have time to read academic papers*, *our work may be stationary*.*” (IDI-HCW012 female)*
**Environmental factors**		
Physical environment	Whether space, ventilation, and facilities are adequate	“*We were fortunate that the number of patients did not outnumber our capacity*, *our equipment*. *So as long as the equipment is sufficient*, *we are OK*. *Patients were very well-taken care off*. *I could not imagine what would happen if the patients outnumbered the negative pressure rooms we have*. *We might be in trouble … even though we are prepared*, *there could be situations that might put us at risk*” (*IDI-HCW010 male*) *“COVID was very dangerous because it could be airborne*, *so we can easily get infected*. *Wearing a mask is a must*, *best if it is N95*, *and if possible put extra coverage on yourself*, *even a DIY one*. *For me at the [virology] lab*, *after unboxing COVID samples*, *we need to take extra caution … discarding all the masks*, *PPE*, *and testing materials every time*.*” (IDI-HCW001 female)*
Availability of PPE	Whether supply of PPE is adequate	*“We were trained how to use PPE*, *of course*, *but when it happened it was more difficult … especially when we needed to take the PPE off*. *Putting it on was simple because there was help from the staff*. *I learned to take it off in steps … the educational poster on the wall helped a lot … but my eyesight did not*. *After a few days you will get a hold of it and learn not to contaminate yourself*.*” (IDI-HCW011 male)* *“I believe we are ready and have enough experience to face another wave [of transmission]*. *But I am a bit concerned about the equipment … we might not have enough equipment for the second wave*. *For instance*, *if the virus mutated we wouldn’t be able to treat the patients like we could now*. *Most of our equipment is also from other countries*, *so I think in order to be even more ready we should start producing our own equipment*.*” (IDI HCW021 male)*
**Individual factors**		
Individual knowledge	How knowledge of IPC guidelines and their importance influence adherence	*“It’s really important to always do a fit test … not only one time a year but regularly*, *especially when dealing with airborne transmission*. *Tuberculosis*, *for example*. *Fit test for N95*, *which brand or type*, *you need to check and be aware of this to be safe*.*” (IDI-HCW018 female)* *“To be honest*, *I think the COVID-19 ward is quite safe at this time because we already know the patients are positive with COVID*. *The risk is highest when we don’t know whether the patients are infected or not*. *Many OPD and IPD staff were concerned but they could not wear full protection then … those who cared for positive cases are safer because they could fully protect themselves*.*” (IDI-HCW008 female)*
Individual attitudes	How HCW perceive the value of IPC guidelines in protecting them, their families, and patients	*“I just tried to avoid contact with other people; normally I drive a motorcycle to work but since COVID-19 I started to drive a car to work instead*. *Sometimes I avoid returning home and stay at the hospital so that I can make sure that I’m not putting family at risk*. *My mother is pretty old so she will be quite vulnerable to COVID-19*. *Especially during March*, *there were way too many COVID samples back then and we had a lot of work to do*.*”(IDI-HCW004 male*)
Individual beliefs	How HCW’s fears, concerns, and duty of care influence adherence to IPC guidelines	*“There were two cases I provided care for … an old lady and her daughter*. *The old lady was* admitted *first with respiratory symptoms but she reported no contact with a high risk person … we did a PCR test for her and it was positive so we asked her daughter to come in for the test as well*. *At first she was very scared and refused to come*. *I had to convince her on the call to let us send the ambulance to pick her up from her apartment but she refused because she was concerned that the residents in her apartment would know and damage her place*. *In the end she decided to drive herself … the mother had diabetes*, *became severely ill and did not survive … so it’s important that we always ask patients to provide us information so we can help them in time*.*” (IDI-HCW018 female)* *“I feel like every area that a patient touches could become a threat*, *every individual who takes care of the patient directly will be at risk*. *Even taxi driver could be at risk driving the patient here*. *As for laboratory staff*, *we don’t meet the patient directly but we take care of all the samples so we need to be very careful not to spill them*.*” (IDI-HCW022 female)*

### Knowledge, perceptions, and concerns of HCWs regarding risk of COVID-19 transmission

When asked about their perceptions about COVID-19 transmission, risk related to the roles of participants were highlighted to most influence HCW’s adherence to IPC guideline. All participants were aware of their risk of COVID-19 transmission at work; however the risk was perceived to vary among different units or staff roles in the hospital. Participants described that those assigned to treatment of COVID-19 patients were at increased risk. Nurse participants working in the outpatient department (OPD) perceived that they might be at higher risk than the in-patient department (IPD) staff because they encountered more patients, either suspected or confirmed, and provided care for those patients regardless of their known status. Virology Laboratory staff who worked with COVID-19 samples reported high awareness of their risk but described that they are more prepared to protect themselves because of previous training and experience working with infectious disease samples.

Perceptions towards transmission risks also varied according to the assigned locations of HCWs within the hospital. Most participants described a temporary OPD that was previously set up to screen and test suspected COVID-19 patients was somewhat unsuitable; they perceived an outdoor acute respiratory infection clinic (ARIC) to have lower transmission risk. One OPD nurse participant compared risk of transmission between IPD and OPD whereby the latter was perceived to have higher risk of transmission for general staff and visitors. One IPC participant respondent described IPD to have higher risk for antimicrobial resistance as there were more patients with clinical symptoms who might receive treatment. Areas with high density and movement of people in the hospital such as waiting areas, screening points, and cafeteria, were also perceived to be of increased risk.

Concerns regarding the transmission risk related to certain medical procedures producing droplets and/or aerosols. This included performing intubation, suction, or resuscitation for patients in OPD and emergency units. A medical doctor participant compared risk of, and prevention used for, COVID-19 to tuberculosis in healthcare settings, for which the former is perceived to be likely transmitted by droplets and the latter by aerosols. Two laboratory staff participants also mentioned that they were primarily concerned about probable contamination of COVID-19 sample delivery packages as they were delivered from multiple sites and might not be properly wrapped and boxed.

In addition to challenges regarding potential for contracting COVID-19 infection themselves, nine nurse participants also expressed concern about subsequently infecting their family members, others in the hospital, and others they encountered, such as on public transport users or neighbours. One participant mentioned feeling distressed from reduced interaction with a close neighbour who was aware of her work at the hospital. Some however argued that public spaces with high human density, such as public transport, markets or department stores, are of higher risk than the healthcare facility as the latter was better screened and managed.

Beyond infection risk, nurse and laboratory staff participants also expressed concern about contingent cost from performing their work related to COVID-19. A few respondents described avoiding the use of public transport and needing to pay more for a private vehicle. One laboratory staff mentioned renting accommodation close by to avoid traveling and reduce the risk of infecting his family members at home. The majority of participants described loss of their private or personal time, particularly with their families. One nurse participant described sending their children to stay temporarily with their parents. Some participants described receiving compensation for their occupational risk from the government and hospital to compensate for their increased risk and additional working hours.

To address some of these concerns, participants discussed the importance of cooperation from others including co-workers, patients, and visitors to effectively implement the IPC measures. Those who provided direct care for patients reported feeling anxious and needing to isolate themselves from colleagues and family members for fear of inadvertently transmitting the disease to them. Nurse participants who were tasked with caring for patients with confirmed COVID-19 and/or worked in a COVID-19 ward mentioned having to self-quarantine for 14 days after their shift to self-monitor and reduce the risk of transmission to their co-workers when returning to their ward.

Some were also challenged by patients when they failed to report their risk of COVID-19 infection, either because they were unaware of their own risk or they felt stigmatized to disclose this information to the staff during screening (or both). For example, two nurse participants described that they and her colleagues were not informed that their patient had contact with a high-risk person, consequently staff implemented a minimal level of prevention. Nurse participants described that this caused delay in giving the right treatment to the patient as well as increasing the transmission risk for the patient and the staff themselves. Some also discussed how the patient might have received more appropriate care and experienced a quicker recovery if she had reported her travel and contact history when initially assessed. For another patient, staff described how they only agreed to disclose his information to the doctor in a closed room to maintain his privacy.

To provide quality care, nurse participants highlighted that their most crucial role was to provide accurate information to patients with a positive COVID-19 test in a clear and empathetic way. Making notes of those conversations with patients was described as useful to keep track of the patient’s concerns and emotional state. This was also mentioned as a way to provide patients with better care and to substitute for limited contact staff may have with patients. Participants also reported doing so in different languages, particularly in Chinese and English, in the early phase of the pandemic before state quarantine was implemented. IPC nurses described that they were aware of the importance of these tasks; however, they felt overwhelmed by the number of phone calls and were unable to continue their training work. Setting up a call centre with staff tasked to professionally communicate and allocate time for the task was mentioned to be a solution preferred by the staff.

The IPC team reported that managing the concerns of HCW were highly challenging, particularly at the early stage. They described COVID-19 as an emerging disease and compared it to the early years of the AIDS pandemic when the disease was perceived to be unknown and stigmatizing. A few staff who had had prior experience with MERS described that they felt prepared for this pandemic; however, some respondents felt that the training on PPE could be provided for a wider group of staff because presumptive COVID-19 patients may be encountered by general OPD nurses and staff. Training targeting non-medical staff was also suggested to be important because they also had risk of exposure in the hospital setting. These included cleaners, receptionists, and security guards who may also not be Thai nationals.

### Availability of PPE and other prevention measures

Although the availability of PPE and measures was emphasized, all participants, especially IPC nurse participants, as crucial to motivate HCW to adhere to guidelines, a majority of participants described that their practice was influenced by its ease of use and HCWs’ readiness. Many COVID-19 prevention measures HCWs reported using were N95 and surgical face masks, face shields, hand washing, PPE, screening for risk factors and symptoms (by taking temperature and/or contact history), social or physical distancing, ventilation, use of negative pressure rooms, and self-quarantine or isolation. Participants discussed how wearing face masks and washing their hands were the most important and practiced measures when working in the hospital. PPE was identified as crucial for doctors and nurses when testing suspected cases or caring for confirmed patients, and for laboratory staff when handling samples. Use of respirators was reported to be essential for anaesthetists and staff in the emergency room (ER) to perform medical procedures. All categories of staff reported these personal protection measures made them feel safer at work.

Several reasons for their use and non-use of COVID-19 prevention measures were reported. Most participants described having high awareness about using the prevention measures and having received appropriate guidance on how to apply them according to their roles. Challenges of using certain measures were described by medical staff participant; for example nurses described taking off the PPE as being a difficult process and they needed help from a colleague to properly remove the equipment and avoid self-contamination. One participant reported that the appropriate use of PPE may depend on experience of the staff and the material of the PPE; material with a harder texture was perceived as being more difficult to remove properly. For masks, participants described the importance of doing a fit test once or twice a year to ensure correct use; some mentioned also using plastic tape to ensure full protection from a mask. A few participants reported that wearing an N95 mask and face shield reduced their ability to see when performing certain medical procedures such as injection or drawing blood. A few participants mentioned that they were unsure whether ultraviolet light can disinfect face masks and for how long.

When faced with inadequate equipment, nurse participants mentioned prolonging use of N95 and surgical masks, purchasing masks for their own use, wearing two masks in a double layer, and use of ultraviolet light to disinfect masks. Hospital telephones or chat applications were used to reduce the time physically interacting with patients and colleagues and reduce the risk from contaminated paper-based documents. An acrylic box used to cover a patient’s head and neck was mentioned to be effective and resource-efficient when performing intubation, resulting in less use of masks and face shields during a shortage. Instructions on how to use PPE were provided to staff so that they were able to perform their tasks; nurses mentioned that a newly established swab unit in the ARIC had helped reduce their need to use PPE when testing for COVID-19.

During the pandemic, donations was described as an alternative to supply necessary material and equipment, and for other additional support such as meals. Participants reported that these had helped to alleviate the period of supply shortage, although important equipment such as masks and PPE needed to be assessed for quality before use. Other measures, such as rescheduling appointments with non-emergency patients were implemented to reduce the number of visitors in the facility. OPD nurses also described specifically designating the task of caring for patients with suspected COVID-19 to one staff per shift or unit to minimize staff’s contact with the patients and therefore reduce the risks for other patients receiving care in the unit.

When asked about the long-term plan for IPC, IPC nurse participants mentioned that keeping the prevention equipment available is important to prepare the staff and alleviate their concerns. IPC nurses also described that demonstrating use of appropriate prevention measures and helping to care for confirmed patients made staff feel more confident and prepared to take on the job, and was a way to give them moral support. In addition to the use of prevention measures, the nurses also reported that HCWs need to monitor themselves under the current working circumstances and should report for testing if they had symptoms, to avoid risk of transmission and reduce concerns among staff. One IPC nurse participant also suggested increasing awareness about some prevention measures, such as correct elevator use, social distancing and hand hygiene, in the hospital.

### Safety of physical environment

Beyond availability, participants also described their use and non-use of certain measures such as masks as being related to the hospital infrastructure. For example, staff stationed at an ad-hoc outdoor ARIC found it uncomfortable to wear a face mask and a face shield for long hours in hot and humid weather. A few nurses also mentioned that it was difficult to communicate with patients through the mask, face shield, and an acrylic barrier. Use of telecommunication was described by most staff to be effective for interacting with patients while keeping distance; some however mentioned that they communicated less effectively with patients given the physical barriers. One participant also reported observing a patient feeling uncomfortable in an outdoor waiting area for an extended period of time. Nevertheless, better ventilation at the outdoor ARIC was perceived to be positive by an IPC nurse who expressed concern about overcrowded, indoor environments in the hospital.

Cleaning and sanitising the environment were described to be necessary measures in the hospital, particularly for units which have suspected and confirmed COVID-19 patients. Nurse participants mentioned a regular schedule for cleaning medical and auxiliary areas and changing patient’s bed sheets. Four nurses in the ARIC who participated in the study reported ventilation to be among the most important measures, especially during periods when a high volume of patients visited the clinic for COVID-19 testing. However, participants described inadequate space for staff to socially distance themselves at work, particularly where more staff worked full-time in a small area. Some also mentioned that patients were sometimes unable to maintain two-metre physical distancing in a small or crowded space such as a waiting area or restroom. In addition, participants discussed a lack of necessary material and equipment such as N95 masks and PPE, especially during the first few weeks of the pandemic. During shortages of protective equipment, participants described that high-risk HCW were prioritised, specifically those who had direct contact with confirmed patients.

### Communication about IPC guideline and availability of training programme

The majority of the interviewed staff felt they were well informed about the information regarding COVID-19 disease and had received necessary training on infection prevention and control. Interviews with IPC nurses identified that prioritised training included fit testing for masks and powered air purifying respirators (PAPR), for which only the emergency unit was usually prepared; training was provided to other units after the initial outbreak. Training on the use of PPE was also discussed; respondents described that the terms used to identify which types of PPE were needed had been adapted for COVID-19 related tasks. Formal PPE training for ARIC staff was prioritized. Laboratory staff described that they had received multiple training sessions as part of their job, and hence felt that they were well equipped to safely carry out COVID-19 related tasks.

For this training, nurse participants described the benefit of different learning tools, such as educational videos and posters. This was described as helping remind staff about the steps for how to properly don or doff PPE; thus reducing their risk and anxiety about self-contamination.

### Management support

Collaboration among units in the hospital was reported to be an important factor to carry out this training. Participants described how various HWCs contributed to each part of COVID-19 training: medical doctors provided knowledge about the disease and how it is transmitted and demonstrated how to do a swab test; IPC nurses provided guidance on how to use the PPE. IPC nurses also described working with the virology laboratory to determine how swab samples should be collected and packaged before sending them for analysis.

Communication channels among HCWs were set up to keep staff informed, however, participants sometimes felt exhausted from changes in updated guidance and recommended practice, particularly early in the pandemic. Participants described being aware of the changing nature of knowledge and practice for a newly emerging disease. Some senior staff emphasized giving clear instructions and updated information as important ways to avoid fatigue and confusion at work. IPC nurses also described simplifying and shortening some guidelines to make them more accessible and easier to understand.

Interviews with IPC nurse found that updating protocols contingent on new information about the disease was a challenge; for example, what symptoms should trigger screening or which countries are high risk. Respondents described the importance of precision when screening eligibility of suspected patients for government-subsidized testing. This had caused difficulty for OPD staff who might not be aware of these constantly-updating criteria, such as addition of high-risk symptoms or countries. The nurse participant who was tasked to manage the updated criteria for case investigation reported felt pressured to give constant guidance to other hospital staff and monitor their adherence to the guideline in the beginning.

To support HCWs in practicing IPC measures, performing a demonstration of the written guidelines enabled staff to get comfortable with the protocols and steps such as screening and triage, and using PPE, were used by the IPC team. Interviews with IPC nurses described that this type of demonstration enabled staff to practice in, and be prepared for, real-life situations. One participant also mentioned that this allowed staff to work together to adapt the guidelines into practice for the context of each ward or unit, for example, determining specific tasks and the number of staff needed based on the resources and capacity.

## Discussion

This study used in-depth interviews to explore the perceptions of healthcare workers about perceived risk, use of prevention measures, and implementation of IPC measures to prevent COVID-19 transmission in a hospital setting in Thailand. Our findings identified the risks and concerns of healthcare workers at their work, the need for adequate training and appropriate protection equipment, and the facilitators and barriers to IPC measures in the hospital setting.

### Perception of the risk of COVID-19 transmission

Our findings show that HCWs were aware of and used prevention practices in healthcare facilities; however, these were not always easy to implement in daily work routines. Interview participants perceived their risk in healthcare settings with regards to their roles. Anaesthetists and ER staff were perceived to be at an increased risk compared to other medical staff because of the medical procedures they performed on both confirmed and unconfirmed patients. Similarly, a retrospective analysis of occupational data to determine the differential risk of COVID-19 by profession showed that healthcare workers involved in procedures which generate aerosols had the highest occupational risk from COVID-19 [21]. In our study, some respondents also described the risk based on location in the hospital and how it may affect their level of protection used; risk of infection was perceived to be higher in an outpatient unit because the unit provided care for a mixed group of patients whose COVID-19 status was unknown. In addition, their use of masks, physical barriers made of plastic or acrylic, and telecommunication have reduced their ability to provide empathetic care to the patients. This has been seen elsewhere in Thailand where one nurse assistant infected from performing routine medical care on a dengue patient who was later diagnosed with COVID-19 [22]. For tuberculosis, the use of masks and respirators had alienating or depersonalizing effects on medical staff working in outpatient departments, HIV clinics, medical wards and tuberculosis clinics in Uganda [23].

Similar to our findings, several concerns about infection and transmission risk of COVID-19 among healthcare workers were identified elsewhere. In Saudi Arabia, medical staff were concerned about limited access to appropriate PPE, testing, up-to-date information and communication, ability to provide competent medical care if HCWs were deployed to a new area (such as non-ICU nurses having to function as ICU nurses), and increased support for other personal needs (such as meals, accommodation and transportation) [24]. Being exposed to COVID-19 at work and transmitting it to those at home were the main causes of anxiety among HCWs in the United States [25]. A multi-country qualitative study found that this is especially highlighted in Thailand and Malaysia where the workers live within a multigenerational household with elderly relatives [26]. HCWs may also be negatively affected by economic and social impacts from COVID-19 public health measures [27]: in Vietnam, higher cost of living and decreasing income were the main causes of concern for frontline HCWs [28]. This implies that HCWs and other staff in healthcare facilities should be prioritised for prevention interventions, including COVID-19 vaccination, to reduce their risk of infection and prevent transmission in healthcare settings [2]. A provision of support package on well-being [29] and mental health support to HCWs [30, 31] were recommended to help HCW cope with increased stress, anxiety, depressive symptoms and enhance the capacity of HCWs during the pandemic. This is particularly necessary for those in low-resource settings in this region [28], including community-based facility workers in Cambodia [32], Thailand [33], Vietnam [34].

### Use of COVID-19 prevention measures and challenges

As identified by respondents, insufficient physical distancing between patients and co-workers, and indoor settings, were identified as among the most important risk factors by healthcare workers in Europe and the UK [35]. Participants also experienced discomfort from the use of N95 masks, face shields, and coveralls, especially when used outdoors in hot and humid weather. In the UK, use of PPE has been shown to cause heat stress and may negatively affect performance, safety and well-being of HCWs during pandemics [36]. A study on perceptions and experiences of HCWs in the US reporting several IPC challenges in the context of COVID-19 suggested that the adaptation of physical space and working environment was crucial in response to some implementation measures, including weather conditions and social distancing [37]. 2021 study on an outbreak investigation reporting transmission among HCWs at a quarantine facility in Thailand suggested use of thorough prevention measures and setting up private living quarters to reduce exposure risk for HCWs whilst performing their clinical work [38]. In Malaysia, HCWs were also found to be particularly at risk at work when COVID-19 was not suspected in patients and insufficient PPE was worn [39], recommending that occupational health check-ups should be conducted to obtain information about the epidemiology of COVID-19 among HCW. Employment of mobile phone application technology to assist patients to self-identify their symptoms was also found to be effective in a Thai hospital setting [40].

Insufficient supply of prevention materials is a barrier to adequate or appropriate use of COVID-19 prevention measures, such as N95 masks and PPE. From the interviews, the acute shortages in medical supplies like PPE and equipment such as ventilators, were reported to be one of the main challenges early during the pandemic. Participants reported using coping strategies in using them, however, these strategies may reduce the preventive effectiveness or increase chances for self-contamination. IPC nurse participants highlighted the significance of improving the management of the supply to ensure that all medical professionals have enough resources to do their jobs as well as protect themselves from the disease. In many countries, hospitals faced limited operational capacity during the beginning of the pandemic due to a large demand shock triggered by acute need for healthcare supplies and health equipment, particularly in low-income countries [41]. In Thailand, 2020 survey among HCWs in public and private hospitals suggested that adequate supply of PPE and emergency preparedness policies need to be put in place to alleviate concerns and anxiety of HCWs during the pandemic without compromising the safety of workers and patients [42].

### Implementation of infection prevention and control measures

The findings also show that training is crucial to ensure that staff are capable and familiar with practicing IPC measures. Medical staff participants highlighted that training should also include auxiliary staff who are not healthcare workers, such as cleaners, because they often assist in cleaning contaminated areas in the facility. More importantly, IPC nurses emphasized the importance of motivating HCWs to implement the prevention measures; support from and collective adherence to such practices among trainers and other colleagues are crucial to positively influence HCWs’ motivation. A rapid evidence synthesis on adherence to IPC guidelines for respiratory infectious diseases among HCW also highlighted the need to include all staff including cleaning and kitchen staff in health facilities when implementing such measures [19]. In Thailand, a recent national survey reporting overuse and underuse of PPE among HCWs during the pandemic in Thailand highlighted that training programmes should be provided and continue to ensure appropriate PPE practice in healthcare settings [43]. In addition, training HCWs on contact tracing and testing among high-risk individuals, supported by the use of a mobile application for reporting, was found to help mitigate COVID-19 transmission in Cambodia [44]. Knowledge about the severity of the disease increased Vietnamese frontline workers willingness to be vaccinated against COVID-19 [45]. Adequate training and appropriate provision of PPE are therefore critical to encourage implementation of prevention measures and reduce differential access to adequate PPE.

From the interviews, HCWs valued clear and comprehensive guidelines to be able to do their work effectively; however, some respondents reported feeling overwhelmed by multiple tasks and practices during the early spread. IPC nurse participants also highlighted that ambiguous and repeatedly changing guidelines may lead to additional workload and work fatigue among HCWs because they needed to take PPE on and off, do additional cleaning, or rearrange their staff and physical space in their unit. Studies on the 2015 MERS outbreak in South Korea also showed that ambiguous and frequently changing guidelines [46], and nonstandardised protocols [47] resulted in HCW’s confusion and burnout. Practical steps taken by IPC nurses suggested that involving staff in adapting the guidelines to the context of each unit, and communicating them in an accessible and transparent manner are effective measures to encourage HCW’s adherence to the guidelines.

### Strengths and limitations

To our knowledge, this is the first study that has used qualitative research methods to specifically address perceptions of healthcare workers during the COVID-19 pandemic in a hospital setting in Thailand. The findings are mainly drawn from self-reported information and might be subject to desirability bias, however, observations and a previous survey [13] provided additional information in the context of healthcare setting and on sensitive topics, such as anxiety. Most participants were female, which reflects the proportion of female nurses in the health facility, and were a diverse group of medical staff, drawn from a range of units and roles. Two non-medical staff were interviewed; however, findings regarding their perception may be less diverse because the study did not include other groups of staff such as cleaning and security personnel. High awareness and preparedness of participants in the study may also be related to the advanced health facility they were working in, which may limit the generalisability of the findings to other facilities. Further research should ideally explore the experience of HCWs in a variety of health facilities, particularly in resource limited settings.

To maintain trustworthiness of the study, it was ensured that interview data were transcribed and read by two researchers (MJ and PC) who are independent from the hospital and that their responses were anonymized and de-identified prior to analysis. Although researchers may have personal (a patient in the hospital) and/or professional (working at the hospital) experiences with the hospital, there was no clear bias at the time of the study. Data saturation was employed to ensure that the interview data is sufficient to described the target topics in depth. Having two researchers transcribe and translate the interviews before coding them line-by-line using software reduced the confirmation bias that may arise from interpretation during the data analysis process. Interview transcripts and coded data were also stored systematically to reduce any potential bias that may arise from individual coding and interpretation by a single researcher. In addition, the findings benefit from additional information from previous KAP study [13]; however triangulation of the findings is limited, and the findings of this study derived wholly from the qualitative interviews.

Implications of the findings for policymakers are summarised in Table 3.

**Table 3:** Implications for policymakers on COVID-19 prevention in healthcare settings.

1. Staff should be prioritised for prevention interventions, including vaccination, to reduce their risk of infection and prevent transmission in healthcare settings 2. The physical environment in may need to be adapted to permit adequate physical distancing 3. Supply of prevention materials must be sufficient for all staff to do their jobs safely 4. Training in use of infection prevention and control measures is crucial and must include auxiliary staff e.g. cleaners 5. Support from colleagues and collective adherence to prevention measures by healthcare workers increase motivation 6. Clear and comprehensive guidelines facilitate good prevention practices whereas ambiguity or changing guidance can cause additional work and fatigue

## Conclusion

Healthcare workers perceived themselves as at increased risk of COVID-19 infection in hospital settings during the early phase of the pandemic. Several factors influenced the use of multiple prevention measures: concerns about infection, availability of consumables and equipment, barriers to work performance, and physical limitations in the hospital setting. Adequate training, clear guidelines, streamlined communications, and management support are crucial to encourage appropriate use of, and adherence to, implementation of IPC measures among HCW. Factors to effectively implement prevention measures and practical guidelines may vary between health facilities. Further studies should thus explore the perceptions and experiences of HCW about how best to protect HCW and patients from COVID-19 transmission or other (re) emerging infectious diseases in a variety of types of health facility during the epidemic or pandemic.

## Supporting information

S1 File ISSM COREQ Checklist. (PDF)

S2 File In-depth interview guide. (DOCX)

S3 File Codebook. (DOCX)

## Abbreviation

ER: Emergency Room
HCW: Healthcare worker
ICN: Infection control nurse
IPC: Infection prevention and control
IPD: Inpatient department
KAP: Knowledge, attitude, and practice
OPD: Outpatient department
PPE: Personal protective equipment
WHO: World Health Organization
