# Supplementary material for: Risks and challenges in COVID-19 infection prevention and control in a hospital setting: Perspectives of healthcare workers in Thailand
Source: PLoS One. 2023 Dec 19;18(12):e0267996. doi: 10.1371/journal.pone.0267996 (PMC10729973; doi:10.1371/journal.pone.0267996)
Supplement: S2 File — (DOCX) [file pone.0267996.s002.docx]

**HCW In-depth interview question guide**

| **Topics** | **Questions and probes** |
| --- | --- |
| **1. Background information** | - Livelihood during COVID-19  - Work experience as HCW |
| **2. Tasks/role as HCW** | - What is your role as a healthcare professional?  - What are your tasks related to COVID-19?  - Do you have any problems completing those tasks? |
| **3. Knowledge on the disease** | - Can you tell me about COVID-19?  - How is the disease transmitted, diagnosed and treated?  - Who is at risk of being infected?  - Are there behaviors that create more risk than others?  - Are the any places that create more risk than others?  - Where do you get the information about the disease? |
| **4. Risk factors and behaviours** | - Do you think you are at risk of getting infected?  - Are there a period of time or place that create more risk for you?  - How do you prevent yourself from getting infected?  - What do you need to prevent yourself?  - Do you have other risks or concerns from your work? |
| **5. Infection prevention and control strategy** | - What do you think about the current infection prevention and control for healthcare professionals?  - What do you see as the main challenges for the strategy?  - How can existing strategy be improved?  - Are there problems with implementing the strategy? |
| **6. Conclusions** | - Would you have anything do add?  - Do you have any questions?  - Would you have any suggestions about specific areas of investigation we should consider in future interviews? |
