# Supplementary material for: Risks and challenges in COVID-19 infection prevention and control in a hospital setting: Perspectives of healthcare workers in Thailand
Source: PLoS One. 2023 Dec 19;18(12):e0267996. doi: 10.1371/journal.pone.0267996 (PMC10729973; doi:10.1371/journal.pone.0267996)
Supplement: S3 File — (DOCX) [file pone.0267996.s003.docx]

**Codebook: Covid-19 HCW Thailand Interviews**

| Covid-19 | Practice |
| --- | --- |
| Experience | Adequacy |
| Attitude | Challenge |
| Belief | Anxiety |
| Infection | Cost |
| Hospital | Health |
| Home | Communication |
| Information | Environment |
| Knowledge | Guideline |
| Risks | Training |
| Activities | Support |
| Locations | Task |
| When | Location |
| Who | Outdoor |
| Prevention | Patient |
| Hand wash | Roles |
| Isolation | Auxiliary staff |
| Mask | Medical doctor |
| Others | Nurse OPD |
| PPE | Nurse IPD |
| Screening | Virology lab staff |
| Social distancing | Time |
